# Circulating Tumor Cells Characterization Revealed TIMP1 as a Potential Therapeutic Target in Ovarian Cancer

**DOI:** 10.3390/cells9051218

**Published:** 2020-05-14

**Authors:** Manuel Abreu, Pablo Cabezas-Sainz, Lorena Alonso-Alconada, Alba Ferreirós, Patricia Mondelo-Macía, Ramón Manuel Lago-Lestón, Alicia Abalo, Eva Díaz, Sara Palacios-Zambrano, Alejandro Rojo-Sebastian, Rafael López-López, Laura Sánchez, Gema Moreno-Bueno, Laura Muinelo-Romay

**Affiliations:** 1 Liquid Biopsy Analysis Unit, Translational Medical Oncology (Oncomet), Health Research Institute of Santiago (IDIS), 15706 Santiago de Compostela, Spain; maabreu20@gmail.com (M.A.); patricia_mondelo@hotmail.com (P.M.-M.); Ramon.Manuel.Lago.Leston@sergas.es (R.M.L.-L.); alicia.abalo.pineiro@sergas.es (A.A.); 2 Department of Genetics, University of Santiago de Compostela, Campus de Lugo, 27002 Lugo, Spain; pablo.cabezas@rai.usc.es (P.C.-S.); lauraelena.sanchez@usc.es (L.S.); 3 Nasasbiotech, S.L., Canton Grande 3, 15003 A Coruña, Spain; lorena.alonso@nasasbiotech.com (L.A.-A.); alba.ferreiros@nasasbiotech.com (A.F.); 4 Fundación MD Anderson Internacional, C/Gómez Hemans 2, 28033 Madrid, Spain; eva.diaz@fundacionmdanderson.es (E.D.); arojo@mdanderson.es (A.R.-S.); 5 MD Anderson Cancer Center, C/Arturo Soria 270, 28029 Madrid, Spain; spalacios@mdanderson.es; 6 Translational Medical Oncology (Oncomet), Health Research Institute of Santiago de Compostela (IDIS), Complexo Hospitalario Universitario de Santiago de Compostela (SERGAS), 15706 Santiago de Compostela, Spain; rafa.lopez.lopez@gmail.com; 7 Centro de Investigación Biomédica en Red de Cáncer (CIBERONC), Monforte de Lemos 3-5, 28029 Madrid, Spain; 8 Departamento de Bioquímica, Universidad Autónoma de Madrid (UAM), Instituto de Investigaciones Biomédicas ‘Alberto Sols’ (CSIC-UAM), IdiPaz, Arzobispo Morcillo 4, 28029 Madrid, Spain

**Keywords:** ovarian cancer, circulating tumor cells (CTCs), cell plasticity, TIMP1

## Abstract

Background: Recent studies showed a relevant role of hematogenous spread in ovarian cancer and the interest of circulating tumor cells (CTCs) monitoring as a prognosis marker. The aim of the present study was the characterization of CTCs from ovarian cancer patients, paying special attention to cell plasticity characteristics to better understand the biology of these cells. Methods: CTCs isolation was carried out in 38 patients with advanced high-grade serous ovarian cancer using in parallel CellSearch and an alternative EpCAM-based immunoisolation followed by RT-qPCR analysis to characterize these cells. Results: Epithelial CTCs were found in 21% of patients, being their presence higher in patients with extraperitoneal metastasis. Importantly, this population was characterized by the expression of epithelial markers as *MUC1* and *CK19*, but also by genes associated with mesenchymal and more malignant features as *TIMP1, CXCR4* and the stem markers *CD24* and *CD44*. In addition, we evidenced the relevance of *TIMP1* expression to promote tumor proliferation, suggesting its interest as a therapeutic target. Conclusions: Overall, we evidenced the utility of the molecular characterization of EpCAM^+^ CTCs from advanced ovarian cancer patients to identify biomarkers with potential applicability for disseminated disease detection and as therapeutic targets such as TIMP1.

## 1. Introduction

Ovarian cancer is an important public health issue in Western countries [1]. It is the ninth most common cancer in women, the fifth most frequent cause of cancer among female population and the principal cause of all gynecologic cancer-related deaths with more than 60,000 new cases reported annually in the United States and the European Union [2].

The majority of malignant ovarian tumors (90%) are carcinomas, being high-grade serous ovarian carcinoma (HGSOC) the most common subtype [1]. Importantly, this tumor has a fatal prognosis with an overall 5-year survival rate lower than 40% because most of the patients present advanced stage disease and tumor masses in the abdomen beyond the pelvis at diagnosis [2]. Although response rates to chemotherapy reach up to 80%, the majority of patients cannot be cured [3]. Therefore, there is an important need for new biomarkers to improve the detection and prognosis of this tumor and also a better knowledge about the molecular events leading to tumor progression and the development of resistance to the treatment.

Although the spread of ovarian cancer is mainly intra-peritoneal [4], some studies have demonstrated the presence of circulating tumor cells (CTCs) in ovarian cancer patients [5,6]. CTCs are rare events in the bloodstream originating from primary tumors and metastases and are considered the main cause of distant metastasis [7]. For this reason, besides the value of CTCs counting as a mirror of the tumor burden, the option to characterize this population provides researchers with a unique tool to gain insight into the key molecular events allowing tumor dissemination in patients with ovarian cancer. Even though previous studies have monitored specific populations of CTCs in patients with ovarian cancer, such as ERCC1, EpCAM, HE4 or Her2 positive CTCs to predict the tumor evolution and the therapy response [3,8,9,10], the biology of this circulating population is still quite unexplored.

Here, we aim to evidence the value of CTCs enumeration and characterization to manage ovarian cancer patients and identify new therapeutic targets to treat these patients. For that, EpCAM positive CTCs were immunoisolated in a cohort of 38 patients with ovarian cancer and characterized using a panel of genes related to ovarian cancer aggressiveness and plasticity. Some of these genes (*MUC1*, *CXCR4*, *CK19*, *CD24*, *CD44*, *TIMP1,* and *GAPDH*) were found highly expressed in the CTCs population from the patients. Importantly, one of these genes, *TIMP1,* was studied in more detail to explore its interest as a potential therapeutic target, demonstrating its growth-promoting role.

## 2. Materials and Methods

### 2.1. Patients Inclusion and Samples Collection

A total of 38 patients diagnosed of ovarian cancer at MD Anderson Cancer Center, Madrid, Spain were included in the study (Table 1) from 2014 to 2016. In addition, 20 age-matched healthy women, with an absence of a previous cancer episode, were also included as controls. All participants signed an informed consent specifically approved for this study by the Ethical Committee of the MD Anderson International Foundation, Madrid, Spain and samples were obtained through MD Anderson Foundation Biobank (record number B.0000745, ISCIII National Biobank Record).

Two tubes (7.5 mL) of peripheral blood were obtained from each patient before treatment onset: one EDTA vacutainer (Becton Dickinson, Franklin Lakes, NJ, USA) for CTCs enrichment and characterization, and one CellSave Preservative tube (Menarini, Silicon Biosystems Inc., Huntington Valley, PA, USA), for CellSearch enumeration.

### 2.2. CTCs Enumeration and Characterization

A total volume of 7.5 mL of peripheral blood (collected with CellSave tube, Menarini, Silicon Biosystems Inc., Huntington Valley, PA, USA) were analyzed for CTCs enumeration by the CellSearch System, using CellSearch Epithelial Circulating Tumor Cell Kit (Menarini, Silicon Biosystems Inc., Huntington Valley, PA, USA). EpCAM enriched cells were labelled with phycoerythrin (PE) conjugated anti-cytokeratins (CKs) antibodies, with allophycocyanin (APC) conjugated anti-CD45 antibodies and with 4,6-diamino-2-phenylindole (DAPI) to identify the nucleus. The CellTracks Analyzer (Menarini, Silicon Biosystems Inc., Huntington Valley, PA, USA) was used to acquire digital images of the three different fluorescent dyes using a 12-bit camera, which were reviewed by trained operators in order to determine the CTCs count. CTCs were considered those cells with round-oval shape, DAPI^+^, CD45^−^ and CKs^+^.

Another 7.5 mL of peripheral blood (collected in an EDTA tube) was used for isolation of EpCAM^+^ CTCs using CELLection^TM^ Epithelial Enrich Kit (Life Technologies, Oslo, AS, Norway) according to manufacturer’s instructions. After the enrichment step, CTCs coupled to the magnetic beads were resuspended in 100 μL of RNAlater (Ambion) and stored at −80 °C until RNA extraction and gene expression analysis by real-time PCR performed as previously described [11]. Briefly, RNA was purified with the QIAmp viral RNA mini kit (Qiagen, Hilden, Germany), specifically designed for very low cellularity samples. cDNA was synthesized by using SuperScriptIII chemistry (Invitrogen, Alameda, CA, USA) following manufacturer’s instructions. To further optimize the sensibility of detection, a preamplification step was performed by using the TaqMan PreAmp Master Mix kit (Applied Biosystems, Foster City, CA, USA) with 14 reaction cycles. Pre-amplified products were subjected to TaqMan real-time PCR amplification for 10 candidate genes (*MUC1, CK19, CXCR4, TIMP1, ALDH1, CD24, CD44, GDF1, GAPDH*) using a 7500 real time PCR System (Applied Biosystems, Foster City, CA, USA) and specific TaqMan assays (Table S1). Expression values for each gene were normalized to *CD45* as a marker of non-specific isolation.

### 2.3. Cell Lines

SKOV3, A2780, OV90, and TOV112 cell lines were acquired from the ATCC. The cells were authenticated by STR-profiling according to ATCC guidelines and maintained at 37 °C in a humid atmosphere with 5% CO_2_ and cultured in McCoy’s 5A medium (Gibco, Grand Island, NY, USA) supplemented with 10% foetal bovine serum (FBS) (Gibco, Thermo Fisher, South America) and 1% penicillin-streptomycin (Gibco, Grand Island, NY, USA), until being tested for TIMP1 protein expression. All functional assays were carried out using the tumoral ovarian cancer cell line SKOV3 (HTB-77), which derives from ascites of a patient with ovarian adenocarcinoma.

### 2.4. TIMP1 Silencing

In order to block the expression of *TIMP1* in the SKOV3 cell line, lentiviral particles containing commercial constructs were used to block the translation of the mRNA that gives rise to the protein. Four different shRNAs (TRCN0000052428; TRCN0000052429; TRCN0000299344; TRCN0000303681) (Mission Lentiviral Transduction Particles, Sigma, St. Louis, MO, USA) were used, following the manufacturer’s instructions, employing a multiplicity of infection (MOI) of 10 and Polybrene (Hexadimethrine bromide; Sigma-Aldrich, Milwaukee, WI, USA) at a final concentration of 8 μg/mL. Commercial particles containing a shRNA directed against a sequence not present in mammals (SHC002V, Mission Non-Mammalian shRNA Control Transduction Particles, Sigma, St. Louis, MO, USA) were used as control. The silenced lines were selected in the presence of puromycin (5 μg/mL) and named as SKOV3_SH3 and SKOV_SH4 while the control was named as PLKO. The efficacy of the silencing was confirmed by RT-q-PCR and Western Blot.

### 2.5. Gene Expression Assays in Cell Lines

RNA was extracted from cell lines using AllPrep^®^ DNA/RNA/Protein Mini Kit (Qiagen, Hilden, Germany) following the manufacturer’s instructions. RNA quantity was assessed using the NanoDrop spectrophotometer (Thermo Fisher Scientific, Wilmington, DE, USA). Next, cDNA was synthesized with 1 μg of RNA by using SuperScript III chemistry (Invitrogen) following manufacturer’s instructions. cDNA was subjected to TaqMan real-time PCR amplification for *TIMP1* and *GAPDH* gene expression analyses using Taqman assays (Applied Biosystems, Foster City, CA, USA) using a QuantStudio3 real time PCR System (Applied Biosystems, Foster City, CA, USA) (Table S1). Expression values for each gene were normalized to *GAPDH*.

### 2.6. Western Blot

In order to confirm the efficacy of the silencing, WB was performed in 10% acrylamide gels, using anti-TIMP1 antibody 1:150 (AF970; R&D Systems, Minneapolis, MN, USA) to incubate the blots overnight at 4 °C. Afterwards, the blots were incubated with peroxidase-conjugated goat anti-mouse polyclonal secondary antibody (1:1000; SC2005; Santa Cruz Biotechnology, Santa Cruz, CA, USA) for 1 h at room temperature. Signal was detected with an enhanced chemiluminescence kit (Thermo Fisher, Waltham, MA, USA) following the manufacturer’s instructions, and β-actin (1:10000: Cat# Ab3280; Abcam, Cambridge, UK) was used as housekeeping control.

### 2.7. In vitro Functional Assays

To characterize the impact of *TIMP1* knockdown on SKOV3 behaviour proliferation, adhesion, colony formation and invasion assays were performed as described below.

#### 2.7.1. Transwell Migration Assay

In order to evaluate the migratory capacity of SKOV3 and *TIMP1* silenced SKOV3 cells, tests were carried out using transwells with a polycarbonate membrane, with a pore size of 8.0 μm (Corning, NY, USA), in 24-well plates. 5 × 10^4^ cells were seeded in transwells containing 100 μL of serum free culture medium. The wells of the plate were filled with 500 μL of complete culture medium with 20% FBS as chemoattractant. After 24 h of incubation, transwells were placed in a well with 500 μL of trypsin to detach the cells that had passed through the membrane. These cells were labelled using Calcein Acetomethyl Ester (4 μM, Invitrogen, Carlsbad, CA, USA), according to the manufacturer’s instructions. The fluorescence emitted by the cells was measured at 485 nm using a FLUOstar Optima fluorometer (BMG Labtech GmbH, Offenburg, Germany) in 96-well plates.

#### 2.7.2. Proliferation Assay

In order to evaluate the proliferative capacity of SKOV3 and *TIMP1* silenced SKOV3 cell lines, assays were carried out in 96-well plates where 10^4^ cells were seeded per well in 200 μL of complete culture medium with 10% FBS. Once adhered (4 h), time 0 was measured. For this, the culture medium was removed and more medium was added with Alamar Blue (Invitrogen, Carlsbad, CA, USA) in a 1:10 dilution. Cells were then incubated for 3 h at 37 °C and the fluorescence was measured using a FLUOstar Optima fluorometer (BMG Labtech GmbH, Offenburg, Germany) at 544 nm. Subsequently, another plate was measured at 72 h, in order to establish the proliferation ratio.

#### 2.7.3. Colony Formation Assay

In order to evaluate the colony formation capacity of the silenced cell lines, agarose assays (Promega, Madison, WI, USA) were carried out in 96-well agarose coated plates. Briefly, 3 × 10^3^ cells were seeded per well, contained in 100 µL of complete culture medium with 10% FBS, supplemented with agarose at a final concentration of 0.3%. This layer was expected to solidify and 30 µl of complete culture medium was added with 10% FBS above. Colony formation measurement was made using the Alamar Blue method (Invitrogen, Carlsbad, CA, USA) that was added in each well to a final concentration of 10%. After 3 h of incubation at 37 °C, the measurement was carried out with a fluorometer at 544 nm. Measurements were made on time 0 and at 96 h, to determine the colony formation occurred during this time interval.

#### 2.7.4. Collagen Adhesion Assay

In order to evaluate the Collagen adhesion capacity of the silenced cell lines to a collagen matrix, adhesion assays were carried out in 96-well plates. Plates were coated with collagen (Sigma, St. Louis, MO, USA), and 5 × 10^4^ calcein (4 μM; Invitrogen, Carlsbad, CA, USA) stained cells were seeded per well in 100 μL of complete culture medium with 10% FBS. After 45 min of incubation, non-attached cells were washed with PBS (×3). Attached cells fluorescence was measured using FLUOstar Optima (BMG Labtech GmbH, Offenburg, Germany) fluorimeter, in order to determinate the percentage of attached cells.

### 2.8. In Vivo Functional Assays Using Zebrafish Xenograft

#### 2.8.1. Zebrafish Care and Breeding

Adult zebrafish (*Danio rerio*) were maintained in 30 L aquaria with a ratio of 1 fish/L of water, with 14:10 day/night cycle and a temperature of 28.5 °C according to the standard procedures. Zebrafish embryos were obtained mating adult zebrafish in a proportion of two females to one male. All the procedures used in the experiments, fish care and treatment were performed in agreement with the Animal Care and Use Committee of the University of Santiago de Compostela and the standard protocols of Spain (Directive 2012-63-DaUE). Experimental protocols were approved by the Ethical Committee of the University of Santiago de Compostela (15010/2015/001). At the final point of the experiments, zebrafish embryos were euthanized by tricaine overdose.

#### 2.8.2. Xenograft Assays and Image Analysis

For the assays, zebrafish embryos were collected from mating adults and incubated from 0 h to 48 h post fertilization (hpf) at 28.5 °C. At 48 hpf, embryos were anesthetized with 0.003% of tricaine (Sigma, Saint Louis, MO, USA) to perform the injection. Control cell line, SKOV3_PLKO and their respective silenced analogous in *TIMP1* gene (SKOV3_SH3 and SKOV3_SH4) were incubated at 37 °C and 5% CO_2_ before the injection until they reached a confluence of 70%. The three cell lines were trypsinized and concentrated at a rate of 1 million cells/eppendorf/sample. After the concentration, all cell lines were dyed with the lipophilic marker DiI (Invitrogen, Carlsbad, CA, USA) and concentrated again separately in 10 µL of PBS (phosphate buffered saline) with 2% of PVP (Polyvinylpyrrolidone) to avoid cellular aggregation.

Cell injection was carried out using borosilicate needles (1 mm O.D. × 0.75 mm I.D.; World Precision Instruments Inc, Sarasota, FL, USA) and a microinjector (IM-31 Electric Microinjector, Narishige International Limited, London, UK) with an output pressure between 26 kPA and 34 kPA and 30 ms of injection time per injection. Between 150–200 labelled cells were injected into the circulation of the embryo (Duct of Cuvier). After the xenotransplantation, embryos were incubated during 6 days post injection (dpi) at 34 °C in 30 mL Petri dishes with salt de-chlorinated tap water (SDTW). Imaging of the injected embryos were performed at different time points during the incubation (1, 4 and 6 dpi) using a fluorescence stereomicroscope (AZ-100, Nikon, Melville, NY, USA) in order to measure the proliferation, spreading and metastasis capacity of SKOV3 ovarian cell line in each condition tested. Quantifish software (University College London, London, UK) was used to quantify the proliferation of the injected cells as described.

#### 2.8.3. Quantification of Cell Proliferation in Zebrafish Assays

Quantifish software (Quantifish 1, Zenodo, University College London, London, UK) was used to quantify the proliferation of the injected cells by means of fluorescence of the DiI labeled cells in each condition in the region of the caudal hematopoietic tissue (CHT) of the embryos, where the cells metastasize. Images provided at different time points were analysed using Quantifish to obtain the number of positive pixels above a certain threshold and the intensity of the fluorescence. With these parameters, a value of integrated density was obtained allowing to compare different times between images and reaching the proliferation ratio between cell injection and 4/6 dpi.

### 2.9. In Vivo Functional Assays Using Mouse Xenografts

All animals were maintained under specific pathogen-free conditions and all procedures were in accordance with institutional guidelines and approved by the Use Committee for Animal Care from the Universidad de Santiago de Compostela (USC, 15010/2015/001). Mouse xenografts were generated after injection of SKOV3_PLKO and TIMP1 silenced SKOV3_SH3 cell lines in the ovary of 8 weeks female SCID mice by a dorsal incision and exposure of the ovary. Once the orthotopic tumor was palpable, mice were euthanized and tumor were minced into small pieces and placed subcutaneously in the flank of three female SCID mice per group. Tumor growth was measured with a digital caliper, and tumor volume was calculated by the formula *V* = (W2 × L)/2 (*V*: tumor volume; W: tumor width; L: tumor length).

Subcutaneous tumors were fixed and paraffin-embedded for histological analyses. Hematoxylin-eosin (HE) staining was performed to determine the presence of tumor cells using Mayer’s Hematoxylin for 4 min and 0.2% eosin for 1 min. Hematoxylin-eosin staining was performed in the Pathology Department of the University Clinical Hospital of Santiago de Compostela.

### 2.10. Statistical Analysis

The statistical analyses were carried out using the software SPSS 22 for Macintosh (IBM Software Group, Chicago, IL, USA), Excel 2011 for Macintosh (Microsoft Corporation, Redmond, WA, USA) and GraphPad Prism 7.0 for Windows (GraphPad Softwares Inc., San Diego, CA, USA).

The analysis of markers expression levels between groups of patients and controls were carried out by means of Mann-Whitney U test. The same test was used to compare the results of the in vitro assays. Comparisons between groups in the zebrafish in vivo tests were carried out using a two-tailed *t*-test analysis to determine the existence of significant differences. In a first step, the outliers of the data series were identified using the ‘identify outliers’ function of GraphPad software (Rout Method) (GraphPad Softwares Inc., San Diego, CA, USA).

Fisher test (two-sided) was used to determine the association between the clinico-pathological features and the levels of the CTC-markers grouped as high and low based on the cut-off, which classifies the 30% of patients as high levels and the 70% as low levels (percentile 70). The diagnostic accuracy of each of the markers was analysed using ROC (Receiver-Operating Characteristic) curves while survival analyses were carried out using the Kaplan-Meier method. For the survival analyses, the levels of CTC-markers were grouped as high/low as previously described. Overall (OS) and progression-free (PFS) survivals were calculated as the time between blood sample collection and patients’ progression/death or last disease control. Correlation analyses for continuous variables were evaluated using the Pearson Correlation Coefficient, while correlations between categorical variables were analysed using the Chi-Square test.

For all the analyses, a probability lower than 5% was accepted as significant (*p* < 0.05).

## 3. Results

### 3.1. CTCs Enumeration by Cellsearch System was Higher in Patients with a More Aggressive Disease

A total of 38 patients treated for either newly diagnosed (*n* = 25) or recurrent (*n* = 11) ovarian cancer at MD Anderson Cancer Center Madrid were enrolled in this study between 2014 and 2016. Patients characteristics are shown in Table 1. The majority of tumors were classified as high-grade serous carcinomas. Besides, 19 patients (50%) had peritoneal dissemination while 11 (28.9%) showed distant tumor locations, mainly in the liver, at sample collection. On the other side, BRCA mutations were found in 10 patients (26.3%). Regarding the evolution of the patients, it is important to mention that the median PFS and OS since blood sample collection was 22.8 and 29.9 months, respectively.

**Figure 1:**
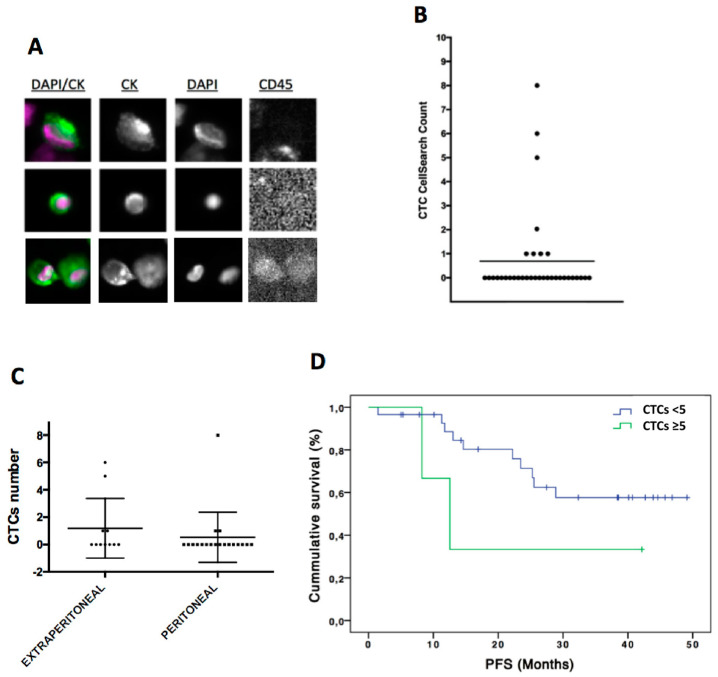
CTCs enumeration by CellSearch system in ovarian cancer patients. (**A**) CTCs images obtained using CellSearch system (round-oval, DAPI^+^, CD45^−^ and CKs^+^ cells were considered as CTCs). (**B**) CTCs count in ovarian cancer patients. (**C**) CTCs count in patients with extra-peritoneal (*n* = 11) and peritoneal metastasis (*n* = 19). (**D**) Kaplan-Meier analysis for PFS grouping patients according to the CTCs count (CTCs number < 5 CTCs versus CTCs number ≥ 5 CTCs). For CTCs < 5 group *n* = 35 while for CTC ≥ 5 CTCs *n* = 3. *p* = 0.18 according to log-Rank test. PFS, progression free survival.

The count of CTCs present in peripheral blood of these 38 patients was evaluated using CellSearch technology. Therefore, only epithelial CTCs expressing EpCAM and CKs were taken into account for the enumeration (Figure 1A). These epithelial CTCs were found in eight patients (21%), in a range between one and eight cells (Figure 1B). Interestingly, the percentage of patients with CTCs was higher in patients diagnosed with extra-peritoneal disease (40%) than in those patients with only local peritoneal metastasis (15.8%), although no significant differences were found between groups (*p* = 0.38 according to Chi-Square test) (Figure 1C). Moreover, the presence of positive (≥1) CTCs was not associated with PFS and OS rates (data not shown). However, patients with a number of CTCs ≥5 (*n* = 3) showed poorer PFS time (median PFS 20.9; CI 3.9–38) in comparison with those with negative count or with <5CTCs (*n* = 33) (median PFS 36.1; CI 29.7–42.5), although no significant differences were found (*p* = 0.18 according to log-Rank test) (Figure 1D). Although showing a trend, these results about the impact of the CTCs count on survival must be interpreted taking into account the low size of the unfavorable groups of patients.

### 3.2. Indirect Quantification of CTCS by RT-qPCR Showed High Accuracy to Detect Cancer Patients

**Figure 2:**
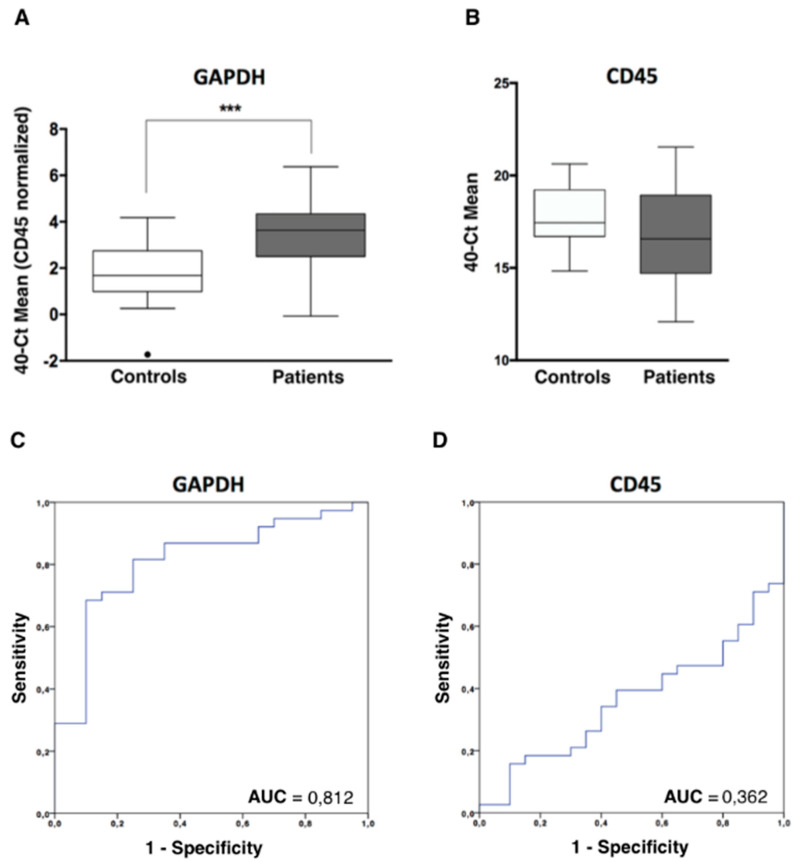
CTCs analysis in ovarian cancer patients by RT-qPCR. (**A**) *GAPDH* expression levels, normalized by *CD45*, in the CTCs fraction of patients and controls, analysed by RT-qPCR (Mann-Whitney U Test, *** *p* < 0.001). (**B**) *CD45* expression levels, in the CTCs fraction of patients and controls, analyzed by RT-qPCR. (**C**,**D**) Power to discriminate patients and controls based on GAPDH and CD45 expression levels, analysed by ROC curves. AUC, area under the curve.

In addition to the CTCs enumeration using CellSearch system, we analyzed and characterized the CTCs population in our cohort of patients by RT-qPCR after the immunoisolation of EpCAM positive CTCs. As we previously reported in other tumor subtypes, firstly we confirmed the presence of an additional circulating population in patients by means of *GAPDH* and *CD45* expression [11,12]. *GAPDH* is a constitutive reference gene, which is characterized by its stable expression and can be correlated with the cellularity of the sample. The levels of *GAPDH* in each sample include both isolated CTCs and non-specifically bound blood cells. In addition, to eliminate this non-specific background of blood cells from each sample, the *CD45* expression was used as a normalizer. As shown in Figure 2A, levels of *GAPDH* in the group of patients were significantly higher than in the group of healthy controls, while the levels of *CD45* did not show significant differences, evidencing the same unspecify isolation of blood cells (Figure 2B). These data indicated that an extra population has been isolated from blood of patients compared to healthy controls and a similar background of blood cells in both groups. Importantly, after ROC analyses, we obtained using *GAPDH* levels an AUROC of 0.81 to discriminate patients from controls (*p* < 0.001) (Figure 2C) while the AUROC curve for CD45 was not discriminative (Figure 2D), as expected.

### 3.3. CTCs Characterization in Ovarian Cancer Patients Evidenced an Important Cell Plasticity

**Figure 3:**
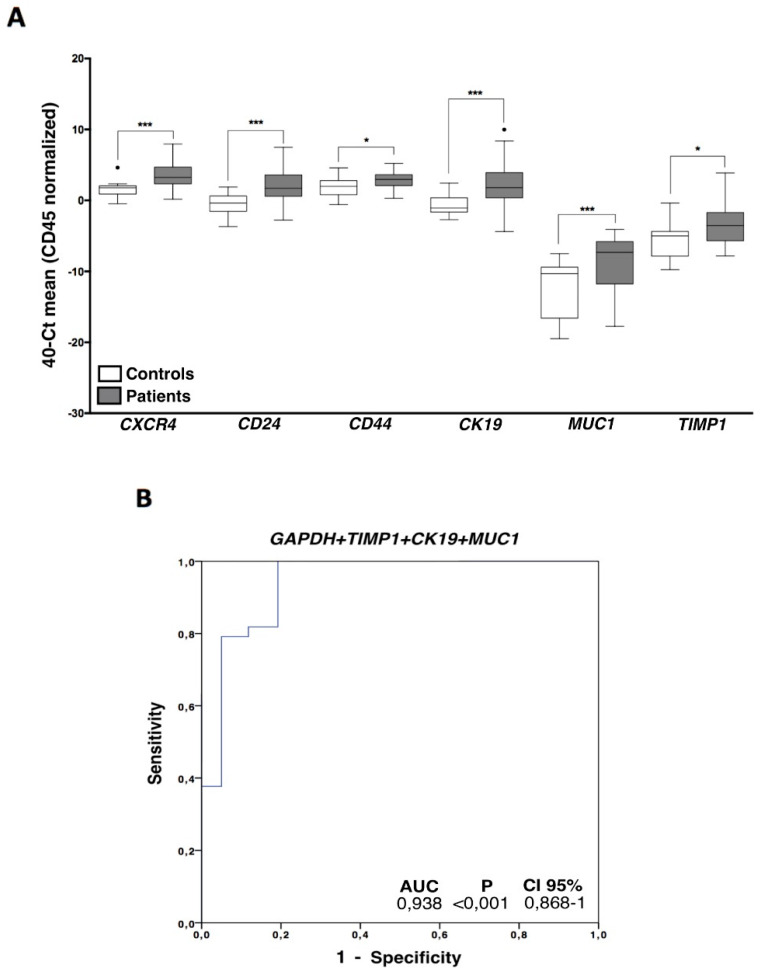
CTCs gene expression characterization. (**A**) Gene expression profile in ovarian cancer CTCs compared with the unspecific isolated fractions in healthy controls. Control group (white boxes), patients’ group (grey boxes) (Mann–Whitney Test; * *p* < 0.05; *** *p* < 0.001). (**B**) Logistic regression ROC model combining *GAPDH, TIMP1, CK19,* and *MUC1* expression levels to discriminate patients and healthy controls. For all the analysis, *n* = 38 patients and *n* = 20 healthy controls.

Once the presence of EpCAM positive CTCs in the blood of patients was confirmed using two methodologies, an expression profile analysis, including a panel of genes related to cell plasticity and ovarian cancer aggressiveness (*MUC1, CK19, CXCR4, TIMP1, ALDH1, CD24, CD44, GDF1*), was carried out. In a first batch of analyzed samples, we observed that *ALDH1* and *GDF1* had no expression or very low expression values and, therefore, we decided to discard them for the following analysis. After analyzing all the samples, we found a CTCs population characterized by epithelial markers expression such as *MUC1* and *CK19,* but these cells also expressed genes associated with mesenchymal and more malignant features such as *TIMP1* and *CXCR4*. On the other hand, *CD24* and *CD44* stem cell markers were also found increased in patient’s samples (Figure 3A, Figure S1A). Importantly, all these genes presented an AUROC to discriminate patients from healthy controls greater than 0.68 (range 0.68–0.81) and with significant *p*-values (Table 2), validating their utility to detect the presence of disseminated disease.

In addition, the diagnostic power of our approach was improved with respect to the use of individualized markers when different markers were combined. Thus, the best panel to detect disseminated disease was obtained when the expressions of *GAPDH*, *TIMP1, CK19,* and *MUC1* were analyzed together (Figure 3B).

Importantly, with this model we were able to determine the presence of disseminated disease in 24 (63%) of the total cohort of 38 patients with a 100% specificity. Therefore, using this combination of markers, 100% of the healthy controls were classified as negative for CTCs count while 63% of the patients presented disseminated disease at sample collection, in comparison with the 21% obtained with the CellSearch system.

On the other hand, when we compared the levels of the CTCs markers and the cell count obtained by CellSearch technology, we observed that patients with positive CTCs count showed significantly higher levels of both *TIMP1* (*p* = 0.04) and *MUC1* (*p* = 0.006), suggesting that conventional (CKs^+^) CTCs identified by the CellSearch system highly express these markers.

### 3.4. TIMP1 Down-Regulation Decreased Tumor Growth In Vitro and In Vivo

**Figure 4:**
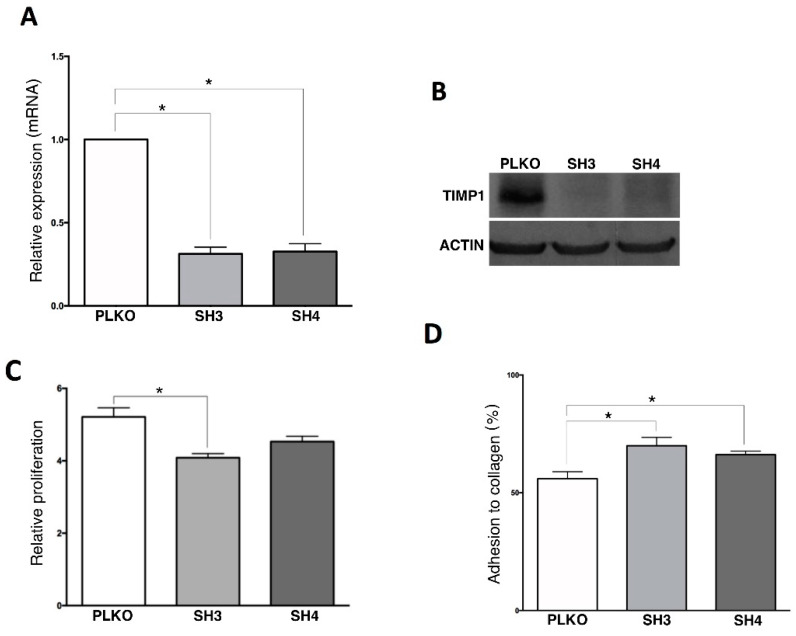
In vitro effect of TIMP1 knock-down on SKOV3 cell line. (**A**) *TIMP1* expression levels analyzed by RT-q-PCR in the control and the TIMP1 knocked-down cell lines. (**B**) TIMP1 protein levels analyzed by Western Blot in the control and the TIMP1 knocked-down cell lines. (**C**,**D**) Results of the Alamar-blue assay to measure cell proliferation and adhesion to collagen matrix. In all experiments, SKOV3_PLKO was the control cell line and TIMP1 knocked-down cell lines were SKOV3_SH3 and SKOV3_SH4. *n*_replica_ = 3 for all the experiments. * *p* < 0.05.

From all markers characterizing the CTCs population in our cohort of patients we selected *TIMP1*, because of its relevance in cancer progression in other cancer subtypes [13,14,15] including triple negative breast cancer where our group found this marker previously [16], for a further in vitro and in vivo characterization. For that, *TIMP1* gene expression was knocked-down in SKOV3 cell line, since this cell line represents serous carcinomas and expresses high levels of the marker (Figure S1B). After stable shRNA transfection, we observed a significant reduction in *TIMP1* expression both at mRNA and protein level in the silenced cell lines (SKOV3_SH3, SKOV3_SH4) in comparison with the control (SKOV3_PLKO) (Figure 4A,B). Furthermore, we also observed a reduction in the proliferation rate in both silenced cells (decrease of 22% and 13%, respectively) with respect to the control cell line (Figure 4C), while no differences were found after TIMP1 down-regulation in colony formation and migration assays (data not shown). On the other side, TIMP1 deficient cells showed higher capacity to adhere to collagen, suggesting a role of this protein in the adhesive characteristics of ovarian cancer cells.

**Figure 5:**
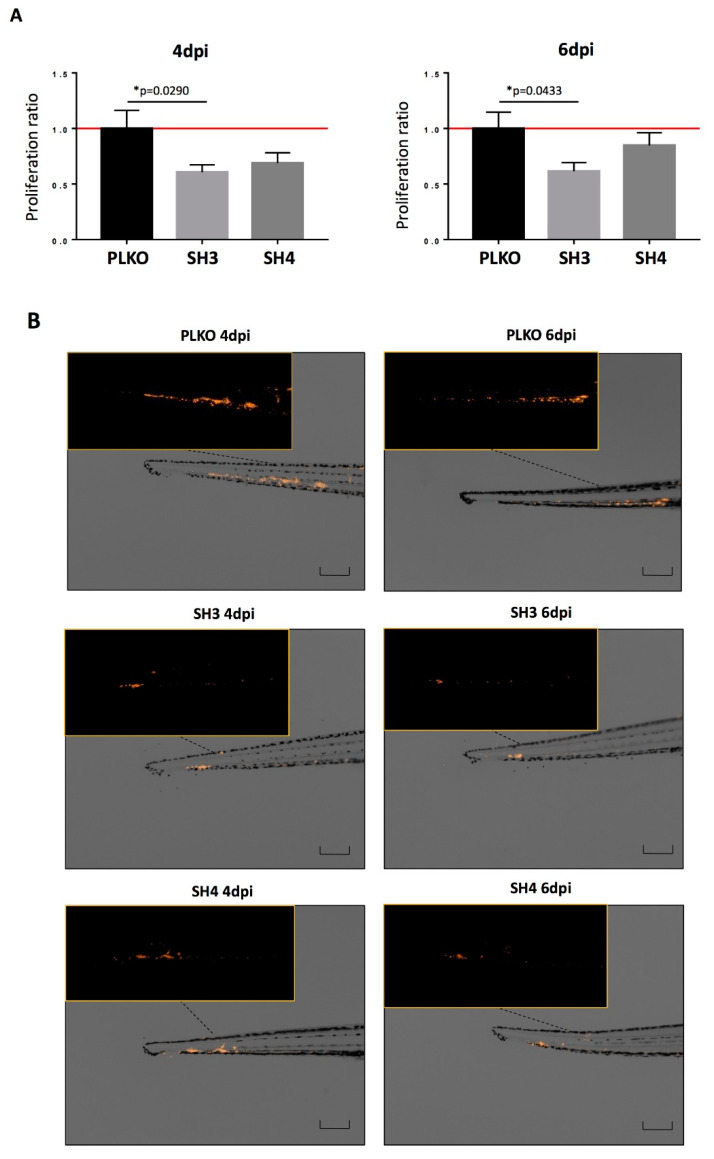
Effect of TIMP1 knock-down in SKOV3 proliferation in zebrafish embryos. (**A**) Representative images of the injected embryos with the different conditions of the TIMP1 silenced cells (SH3 and SH4) and the control cell line (PLKO) at 4 dpi and 6 dpi compared to 1 dpi. Main images are a superposition of a fluorescence image and a bright field image of the same embryo. Fluorescence images are a magnification of the areas marked in the main image. Scale = 250 µm. (**B**) Normalized tumor growth at 4 dpi (left panel): SKOV3_PLKO = 1.00, SKOV3_SH3=0.6072, SKOV3_SH4=0,6898; and 6 dpi (right panel): SKOV3_PLKO = 1.00, SKOV3_SH3 = 0.6155, SKOV3_SH4 = 0.8484 (*n*_replica_ = 15 embryos/condition, *n*_total_ = 45 embryos/condition; * *p* < 0.05 according to two-sided T-test).

In addition to the in vitro assays, we used zebrafish embryos to analyze in vivo the effect of TIMP1 down-regulation on SKOV3 cell line. For that, DiI labeled SKOV3 variants (PLKO, SH3 and SH4) were injected into the circulation of 48 hpf zebrafish embryos to analyze the differences between the silenced cells and the control one in terms of proliferation and metastatic capacity. After injection, incubation of zebrafish embryos was carried out for 6 days at 34 °C. Our findings evidenced a reduction of proliferation in the TIMP1 knocked cells compared to the control cell line (Figure 5A,B). In fact, the proliferation of SKOV3_SH3 in the CHT of the embryos was 40% lower (*p* < 0.05) than the control condition at 4 dpi, and this reduction was maintained at 6 dpi (39% lower, *p* < 0.05). SKOV3_SH4 cells also showed a tendency of growth reduction compared to the control, but not being statistically significant even at day 6 of incubation (Figure 5A).

**Figure 6:**
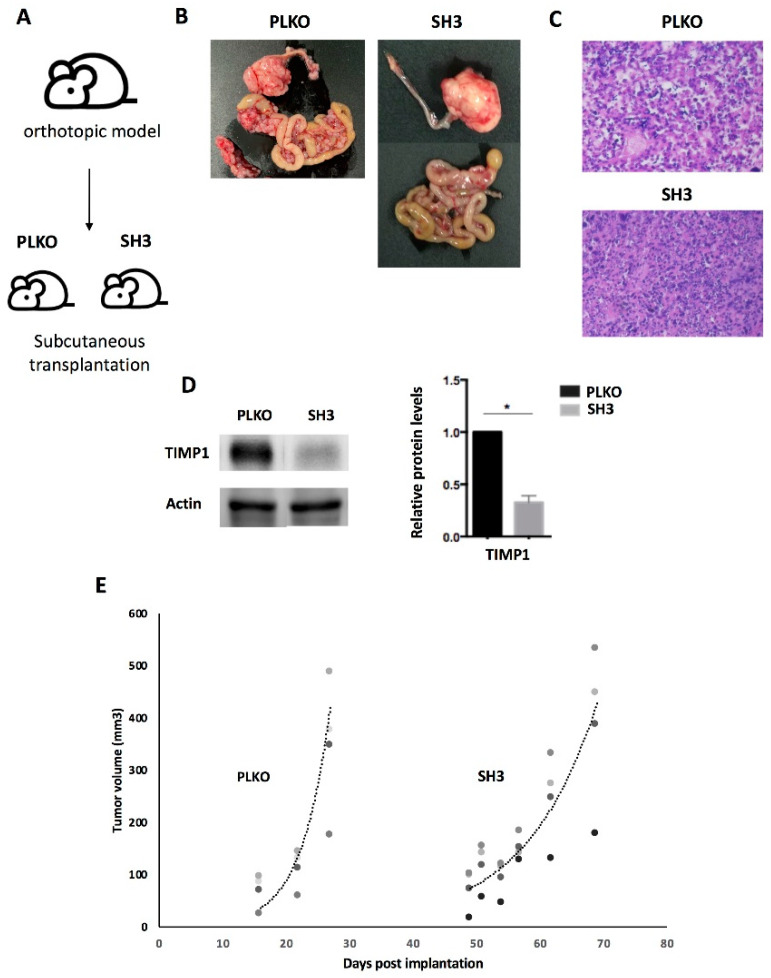
Effect of TIMP1 knock-down in SKOV3 proliferation in a mice model. (**A**) Representative scheme of the assay. The TIMP1 silenced cells (SH3) and the control cell line (PLKO) were injected in the ovary of SCID mice and the generated tumors amplified subcutaneously in the flank of three female SCID mice per group. (**B**) Macroscopic image of tumor generated orthotopically. (**C**) Representative images of hematoxylin-eosin staining of tumors generated in the subcutaneous model (objective 10x). (**D**) Western blot results for TIMP1 protein levels in tumors generated by SKOV3_PLKO and SH3 cell lines. Actin was used as normalizer. (**E**) Dynamic of tumor growth after subcutaneous transplantation, which was slower in the TIMP1 silenced cell line. *n*_replica_ = 3 for the two experimental groups. * *p* < 0.05.

To further validate this impact on cell growth, the behavior of both SKOV3_PLKO and the TIMP1 down-regulated cell line SH3, which showed the highest effect on the zebrafish model, were evaluated in mice. For this, these cells were injected in the ovary of SCID mice and the generated tumors amplified subcutaneously in the flank of three female SCID mice per group. As Figure 6 shows, the tumor growth was lower in the TIMP1 silenced tumors in comparison with the control ones. These results confirmed in vivo the impact of *TIMP1* silencing on the proliferative behavior of our ovarian cancer model.

## 4. Discussion

Circulating tumor cells (CTCs) are mainly responsible for the metastasis formation and have tumor-specific molecular characteristics [7]. These cells’ enumeration has shown to have a prognostic value in patients with different tumors as breast, colorectal, gastric, lung, and prostate cancer [17]. Some studies have revealed the presence of CTCs in patients with ovarian cancer in spite of the fact that the frequency of CTCs is low compared with other solid tumors [18,19,20]. This low detection rate is normally linked to the fact that ovarian cancer shows less hematogenous spread [21] and, therefore, the sensitivity of the isolation and detection strategy applied to catch these cells must be higher. Regarding this limitation, the presence of CTCs in ovarian cancer patients has been associated with an overall worse prognosis and advanced disease stage using different methodologies [5]. On the other side, the characterization of CTCs in ovarian cancer is nowadays considered as a relevant tool to provide researchers with a better picture of the molecular actors leading to hematogenous spread and distant metastasis formation, although it has not been explored much.

Detection rates of CTCs between 18% and 88% have been described depending on the detection method used in patients with FIGO Stage I–IV ovarian cancer [5]. In the present study, we analyzed CTCs levels in patients with high grade ovarian cancer using both direct and indirect quantification strategies after an EpCAM-based immune-enrichment. With CellSearch system, the reference technology for CTCs enumeration, we found CTCs in 21% of patients with a range of 1 to 8 CTCs. However, using an indirect quantification strategy where different transcripts were evaluated by RT-q-PCR after the EpCAM^+^ CTCs immunoisolation, the rate of CTCs positive patients increased until 63%, probably because with this strategy CTCs fragments and also non-conventional CTCs are contributing with more target transcripts, in addition to the conventional epithelial CTCs detected using CellSearch system. In this sense, it is important to remark that CellSearch criteria for CTCs selection only classifies as CTCs those cells with round-oval morphology, positivity for CKs and DAPI and negativity for CD45. Therefore, with our gene expression approach, we evaluated also CTCs with low CKs expression. In fact, it has been established that CTCs are highly plastic, making necessary new approaches to consider CTCs with an epithelial as well as mesenchymal phenotype [22]. Our group successfully employed this gene expression strategy to detect and characterize this circulating population in colorectal, lung, prostate, endometrial, and triple negative breast cancer, providing valuable information about mechanisms behind tumor dissemination and progression [11,12,16,23,24].

In the present study, in order to characterize the CTCs population in our ovarian cancer patients’ cohort, genes previously associated with both epithelial, mesenchymal, stem and aggressive characteristics of ovarian cancer were analyzed. From them, *CK19, MUC1, CD24, CD44, TIMP1,* and *CXCR4* appeared, characterizing the ovarian cancer circulating population. These six CTCs markers showed a strong value to discriminate patients and controls with high sensitivity and specificity, therefore, they constitute an interesting tool for ovarian cancer diagnosis. On the other hand, the levels of these markers were no significantly associated with the patients’ outcome as happened with other clinico-pathological characteristics, probably because of the short follow-up of some patients included in the study.

Going inside the biological meaning of the CTCs panel identified, it is important to remark that *CK19* and *MUC1* expression has been previously described in CTCs from ovarian cancer patients using both antigen-dependent or independent isolation strategies [3,19,25], validating the epithelial origin of these cells. Importantly, the presence of *CD24* and *CD44* in our ovarian CTCs population is in line with the idea that CTCs normally show an important molecular plasticity, expressing both epithelial, mesenchymal and stem markers, which mediate CTCs migration and survival under the pressure of adverse microenvironments and chemotherapy. Nakamura et al. recently demonstrated that *CD24* is highly expressed in primary ovarian carcinoma tissues, being correlated with the presence of lymph node metastasis and representing an independent predictor of survival [26]. On the other side, *CD44* expression has been associated with high-grade and advanced FIGO stage ovarian carcinomas [27]. In addition, overexpression of *CD44* in ovarian cancer cells increases their proliferation, adhesion and invasion capacity [28]. Recent single cell transcriptome data obtained in CTCs from prostate and breast cancer patients demonstrated that these cells express *CD44* [29]. Blassl et al., also analyzed at single CTC level *CD44* transcripts in three patients with ovarian cancer, finding that only a part of the CTCs, those with high expression of mesenchymal markers, were expressing the receptor [30]. Besides, the expression of *CXCR4* detected in ovarian CTCs reinforces the relevance of cell plasticity for the dissemination process. In fact, the levels of this receptor are strongly increased in high-grade serous ovarian carcinomas, the most metastasic subtype [31]. Moreover, *CXCR4*-expressing ovarian cancer cells have more mesenchymal characteristics and more invasive capacity. In accordance, *CXCR4* inhibition has been associated with reduced levels of CTCs and metastasis in ovarian cancer mouse models [31].

Our results also demonstrated that TIMP1 is present in CTCs from patients with ovarian tumors. This tissue metalloproteinase inhibitor is a principal actor for maintaining tissue integrity and has emerged as an important factor implicated in different pathologies including cancer. TIMP1 is known to regulate the activity of MMP9, which promotes tumor migration due to extracellular matrix degradation. Recently, Będkowska et al. described increased levels of TIMP1 in serum from ovarian cancer patients in comparison with healthy controls, as Maatta et al. previously found in 22 cases of serous, mucinous and others malignant ovarian tumors [32,33]. These results were also in line with other studies where TIMP1 circulating levels, together with MMP9, were analyzed using plasma from ovarian cancer patients [34]. Regarding the prognostic value of the metalloproteinase inhibitor, conflicting results have been reported. Some studies failed to demonstrate an impact of TIMP1 tissue levels on ovarian cancer survival rates [35,36], while others described elevated pre-operative or post-chemotherapy serum levels associated with unfavorable clinical outcomes [34,37].

We recently described the expression of *TIMP1* in CTCs from patients with triple negative breast cancer associated with a poor prognosis and we also demonstrated its role for promoting the tumor growth in vitro and in vivo [16]. In fact, TIMP1 was described as a valuable molecular target in several tumors where its role as MMP9 inhibitor as well as promotor of proliferation and angiogenesis has been well characterized [38]. A recent study has also evidenced the increment of TIMP1 expression in platinum-resistance ovarian cancer cells and its interest to predict global patients with advanced tumors [39]. However, its role in ovarian cancer is still quite unclear. Therefore, in our study, we used SKOV3 cell line as a cellular model and down-regulated *TIMP1* expression with the goal of shedding new light on the role of this metalloproteinase in ovarian cancer. Importantly, we observed a decrease in this cell line proliferative capacity in vitro. Additionally, we generated zebrafish xenografts to in vivo analyze the impact of TIMP1 down regulation, finding again a significant reduction of tumor proliferation. The same effect was also observed in a mice model generated with the SKOV3 cell after TIMP1 knock-down. The promotion of cancer proliferation by TIMP1 activity has been described in other tumor types as leukemias, colon, gastric or breast cancer [13,40,41,42], but never until now in ovarian cancer. This promotion was associated with the regulation of different pathways including the axis PI3K/AKT [13,41]. Interestingly, since the axis CXCR4/SDF1 has been described as an important element for the apoptosis inhibition mediated by TIMP1, we hypothesize that both *TIMP1* and *CXCR4* expression in CTCs from ovarian cancer patients could be playing an important role for avoiding apoptosis phenomena. Although the molecular mechanisms behind the role of TIMP1 for ovarian cancer proliferation and dissemination should be studied in more detail, our results open an interesting line of research, indicating its value as a potential therapeutic target.

## 5. Conclusions

Overall, we demonstrated that although ovarian cancer mainly disseminates locally, CTCs count and characterization could provide interesting information to establish a new option for ovarian cancer diagnosis and also to identify the mechanisms leading to ovarian cancer spread. In fact, we found a CTCs signature characterized by cell plasticity in terms of EMT and stem markers that should be validated in a larger cohort of patients as a potential tool for prognosis and therapy monitoring in ovarian cancer patients. Finally, we demonstrated in vitro and in vivo that *TIMP1*, one of the genes characterizing the CTCs population, promotes proliferation of ovarian cancer cells, suggesting its potential as a therapeutic target.

## Figures and Tables

**Table 1:** Patients’ characteristics.

Features		
**Age**	Mean (range)	58 (23–83)
		n (%)
**Stage at diagnosis**	I	1 (2.6%)
	II	5 (13.2%)
	III	21 (55.2%)
	IV	5 (13.2%)
	Unknown	6 (15.8%)
**Status at sample collection**	First diagnosis	25 (65.8%)
	Recurrence	11 (28.9%)
	Unknown	2 (5.3%)
**Metastasis at sample collection**	Peritoneal	19 (50%)
	Extraperitoneal	11 (28.9%)
	Unknown	8 (21%)
**Histology**	Serous	22 (57.9%)
	Endometrioid	3 (7.9%)
	Other	10 (26.3%)
	Unknown	3 (7.9%)
**Grade**	High	36 (94.7%)
	Unknown	2 (5.3%)
***BRCA* status**	Mutant	10 (26.3%)
	Wt	26 (68.4%)
	Unknown	2 (5.3%)
**Under treatment at sample collection**	Yes	9 (23.7%)
	No	29 (76.3%)
**CA125 levels at diagnosis (units/mL)**	>35	24 (63.2%)
	<35	3 (7.9%)
	Unknown	11 (28.9%)
**Recurrence**	PD	12 (31.5%)
	PFS (median months, CI)	22.8 (0.39–49.1)
**Survival**	*Death*	6 (15.8%)
	OS (median months, CI)	29.9 (0.39–49.1)

CI, confidence interval; PD, progression; PFS, progression free survival; OS, overall survival.

**Table 2:** Diagnosis value of the markers analyzed in CTCs.

Marker	AUC ^1^	*p*	CI 95%
*MUC1*	0.812	<0.001	0.703–0.920
*CXCR4*	0.880	<0.001	0.783–0.976
*CK19*	0.826	<0.001	0.718–0.933
*CD24*	0.845	<0.001	0.746–0.945
*CD44*	0.686	0.021	0.540–0.831
*TIMP1*	0.696	0.015	0.552–0.840
*GAPDH*	0.812	<0.001	0.695–0.929

^1^ AUC: area under the curve.

## Supplementary Materials

The following are available online at https://www.mdpi.com/2073-4409/9/5/1218/s1; Figure S1: A, Representative Heatmap of gene expression profile in ovarian cancer CTCs compared with the unspecific isolated fractions in healthy controls. Markers were grouped as high, medium and low according to percentiles 33 and 66. B, TIMP1 expression levels in SKOV3, A2780, OV90 and TOV112 cell lines analysed by Western blot. Actin was used as normalizer; Table S1: Gene expression assays used to characterize the CTCs population.
