# Supplementary material for: Circulating Tumor Cells Characterization Revealed TIMP1 as a Potential Therapeutic Target in Ovarian Cancer
Source: Cells. 2020 May 14;9(5):1218. doi: 10.3390/cells9051218 (PMC7291036; doi:10.3390/cells9051218)
Supplement: Supplementary file 1 [file cells-09-01218-s001.zip › Supplementary Table1.docx]

| **Supplementary Table 1: Gene expression assays** | |
| --- | --- |
| **Gene** | **Taqman assay** |
| *ALDH1* | Hs_00946916_m1 |
| *CD24* | Hs_00273561_s1 |
| *CD44* | Hs_01075861_m1 |
| *CD45* | Hs_00894734_m1 |
| *CXCR4* | Hs_00607978_s1 |
| *GAPDH* | Hs_99999905_m1 |
| *GDF1* | Hs_00242151_m1 |
| *CK19* | Hs_00761767_s1 |
| *MUC1* | Hs_00159357_m1 |
| *TIMP1* | Hs_00171558_m1 |
| *CD133* | Hs_01009250_m1 |
