# Supplementary figures and images for: Circulating Tumor Cells Characterization Revealed TIMP1 as a Potential Therapeutic Target in Ovarian Cancer
Source: Cells. 2020 May 14;9(5):1218. doi: 10.3390/cells9051218 (PMC7291036; doi:10.3390/cells9051218)

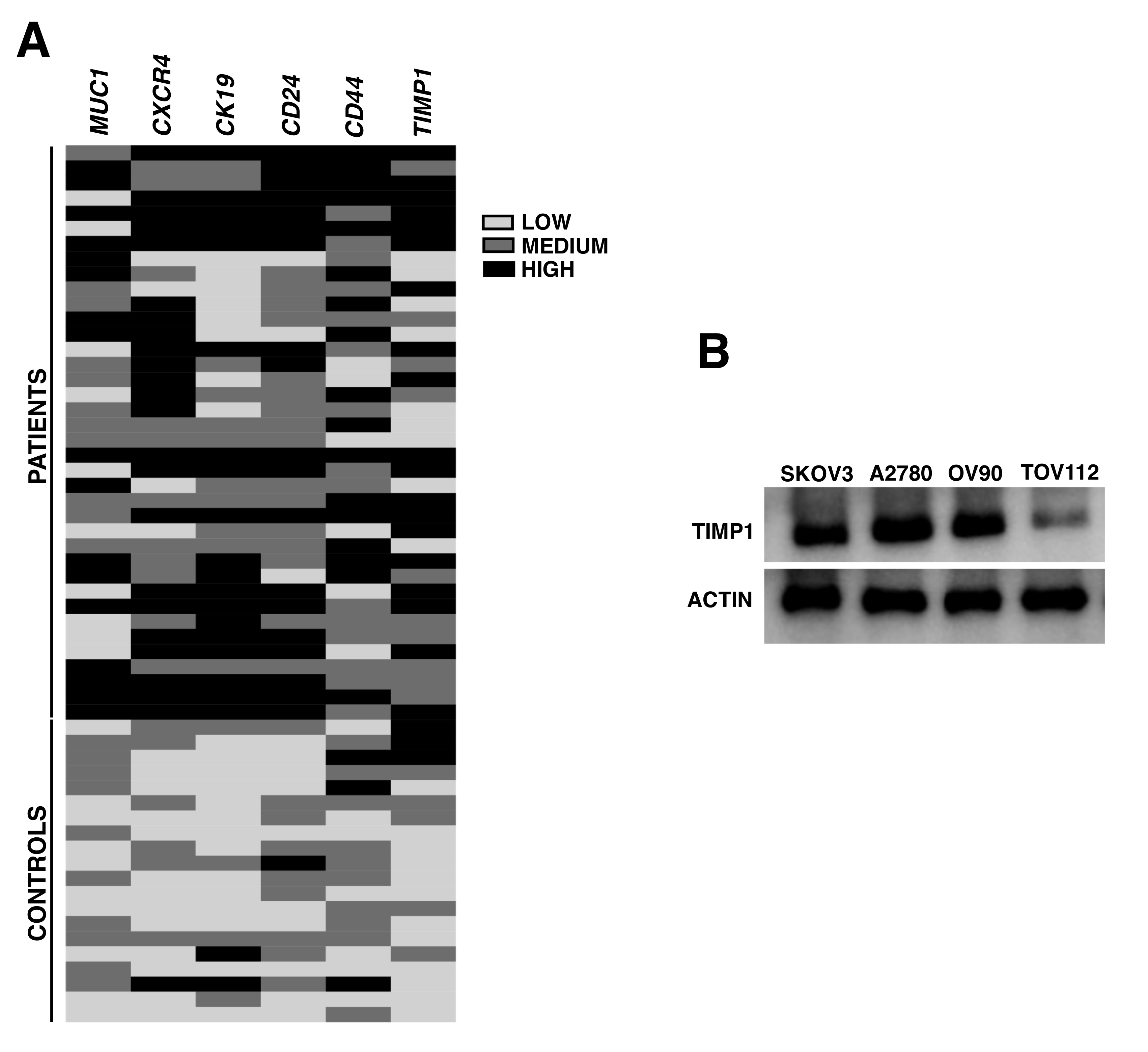

Supplement: Supplementary file 1 [file cells-09-01218-s001.zip › Supplementary Figure 1.tiff]
